# Giant adrenal myelolipoma in a young female patient: a case report

**DOI:** 10.1093/jscr/rjac213

**Published:** 2022-05-28

**Authors:** Moez Rahoui, Yassine Ouanes, Kays Chaker, Kheireddine Mrad Dali, Mokhtar Bibi, Ahmed Sellami, Sami Ben Rhouma, Yassine Nouira

**Affiliations:** Urology Department, La Rabta Hospital, Tunis, Tunisia; Urology Department, La Rabta Hospital, Tunis, Tunisia; Urology Department, La Rabta Hospital, Tunis, Tunisia; Urology Department, La Rabta Hospital, Tunis, Tunisia; Urology Department, La Rabta Hospital, Tunis, Tunisia; Urology Department, La Rabta Hospital, Tunis, Tunisia; Urology Department, La Rabta Hospital, Tunis, Tunisia; Urology Department, La Rabta Hospital, Tunis, Tunisia

## Abstract

Myelolipoma is a rare, benign, non-secreting tumor and its pathophysiology is of metaplasia of the cells of the adrenal cortex into reticuloendothelial cells. Although they are often small and asymptomatic, some cases of giant adrenal myelolipoma cause symptoms such as chronic pain. Few cases of adrenal myelolipoma have been reported in the literature. We present a case of a large right adrenal myelolipoma in a 26-year-old female patient, who presented with an adrenal mass, and discuss the challenges of diagnosis and treatment.

## INTRODUCTION

Adrenal myelolipoma is a rare benign, nonfunctional lesion that is composed of macroscopic fat and mature hematopoietic tissue, resembling bone marrow [1]. A few cases have been reported in the literature. In the past, some tumors were discovered at autopsy, with an incidence ranging from 0.08 to 0.4% [2]. Today, with the progress made in imaging, incidental detection of myelolipoma has become more frequent, constituting up to 10–15% of incidental adrenal masses [1]. Although they are often small and asymptomatic, some cases of giant adrenal myelolipoma cause symptoms such as chronic pain. We report a case of giant right adrenal myelolipoma detected with computed tomography (CT) in a young female patient with right abdominal pain.

## CASE REPORT

**Figure 1:**
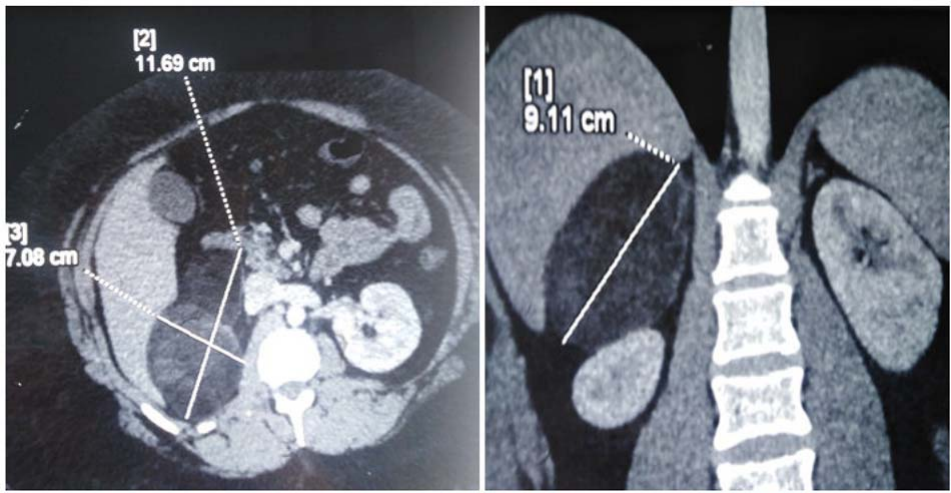
CT scan image showing right adrenal fatty mass.

**Figure 2:**
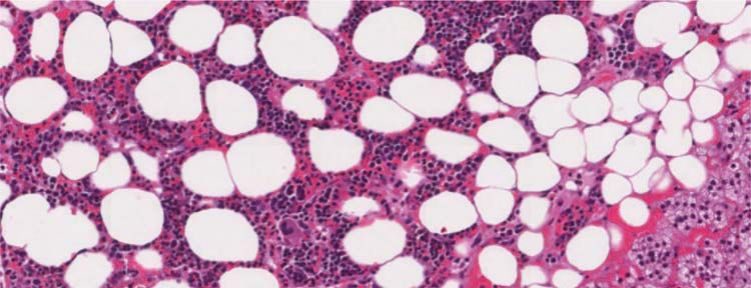
Microscopic appearance of adrenal myelolipoma; typical histological features of a myelolipoma comprising adipose tissue mixed with areas of hematopoietic tissue.

A 26-year-old female patient, presented with right flank pain evolving for 4 months. The clinical examination showed a morbid obesity with a body mass index of 42.8, a pulse at 85 bpm and normal blood pressure of 125/85 mmHg. CT showed a right retroperitoneal mass measuring 11 × 7 cm on the axial plane with a large axis of 9 cm. The mass appeared largely fatty with areas of higher attenuation inside. Density on CT varied between −90 Hounsfield units (HU) peripherally and −30 HU in the center (Fig. 1). Laboratory investigations were normal. A screening hormone test related to the adrenal gland revealed normal plasma catecholamine levels; epinephrine, 0.07 ng/ml (*N*: 0.00–0.10); norepinephrine, 0.28 ng/ml (*N*: 0.10–0.50). Furthermore, detailed 24-h urinalyses during hospitalization showed normal urinary catecholamine levels. The rest of the endocrine exploration was normal. The patient was taken up for exploratory laparotomy. Intraoperatively, a giant solid mass was found arising from the right adrenal gland and was adherent the upper pole of the kidney, duodenum and liver. The patient underwent excision of this adrenal mass. Macroscopically, the mass measured 12 × 8 × 10 cm and was round, well circumscribed and encapsulated. Histologically, the tumor was confirmed as a myelolipoma, and no malignant foci were detected (Fig. 2). The patient had an uneventful recovery and was discharged on the fifth post-operative day. Six months following the surgery the patient had no significant complaints and reported that her right flank pain had since subsided.

## DISCUSSION

Adrenal myelolipoma is a rare benign tumor. Its pathophysiology is thought to be metaplasia of adrenal cortical cells into reticuloendothelial cells in response to infection, chronic stress or degeneration of the adrenal gland [1, 2]. The average age of discovery is around 50 years of age [2]. Generally, incidental discovery can cause symptoms such as chronic pain [2]. Histologically, the tumor is composed of mature fatty tissue associated with normal hematopoietic tissue [3]. Thus, the echogenicity of the tumor depends on the predominance of the fatty or myeloid component. Its diagnosis is made by the CT scan that identifies the fatty contingent [2, 3]. However, these radiological aspects can lead to confusion with angiomyolipoma of the kidney, lipoma and liposarcoma [3]. In these cases, magnetic resonance imaging allows a better characterization of the lesion allowing confirmation of the benign nature of this tumor [3].

In our case, the patient was symptomatic of pain and the dimensions of the adrenal myelolipoma were >4 cm. In addition, the patient presented clinical signs of a hormone-secreting tumor (truncal obesity and abdominal striae), although the hormonal exploration was normal. This patient’s clinical signs and symptoms along with the CT imaging warranted concern for malignancy such as liposarcoma. This was the main indication for surgical treatment.

The treatment of myelolipoma is not clearly defined. Some studies suggest that patients with small lesions should not be treated [4]. However, some authors have proposed surgical treatment [1, 3]. It is suggested that symptomatic tumors or myelolipomas >7 cm should be surgically excised [1]. The removal of the myelolipoma is also indicated when it is compressive, or presenting a hemorrhagic risk [4]. Adrenal myelolipoma generally does not recur, with recurrence-free survival rates of up to 15 years reported in the literature [5]. Although the tumor is benign, surgery may have an important role, especially in symptomatic cases and lesions which cannot be reliably distinguished.

In conclusion, adrenal myelolipoma is a rare entity and is mostly asymptomatic. The diagnostic strategy is the same as for adrenal incidentalomas. The CT scan allows the diagnosis in the majority of cases. The surgical removal of myelolipoma is indicated when it presents a size >7 cm, with compressive symptoms or presenting with bleeding complications.

## CONFLICT OF INTEREST STATEMENT

None declared.

## FUNDING

None.
